# A validation of the ICECAP-O in a population of post-hospitalized older people in the Netherlands

**DOI:** 10.1186/1477-7525-11-57

**Published:** 2013-04-08

**Authors:** Peter Makai, Marc A Koopmanschap, Werner BF Brouwer, Anna AP Nieboer

**Affiliations:** 1 Department of Geriatrics, Radboud University Nijmegen Medical Centre Nijmegen, Renier Postlaan 4, Nijmegen 6525GC, The Netherlands; 2 Institute of Health Policy and Management, Erasmus University Rotterdam Burgemeester, Oudlaan 50, Rotterdam 3062, PA, The Netherlands

**Keywords:** Capability wellbeing, Health-related quality of life, ICECAP-O, Post-hospitalized elderly

## Abstract

**Background:**

Various healthcare and social services may impact not only health, but wellbeing as well. Such effects may be more fully captured by capability-wellbeing instruments than with Health-related Quality of Life (HrQol) instruments. The aim of this study is to validate the ICEpop (Investigating Choice Experiments for the Preferences of Older People) CAPability measure for Older people (ICECAP-O) capability wellbeing instrument in a population of post-hospitalized older people admitted to a hospital 3 months earlier.

**Methods:**

296 post-hospitalized older people in the Netherlands were interviewed 3 months after admission between September 2010 and January 2011. We investigated the convergent validity of the ICECAP-O and overall wellbeing measures (Cantril’s ladder and Social Production Function: Instrument for Level of Well-being (SPF-IL)), as well as with various health measures (EQ5D, Katz-15 Instrumental Activities of Daily Living (IADL) scale, Geriatric Depression Scale (GDS) and the Medical Outcomes Study Short form (SF-20) social functioning dimension). Additionally, we assessed discriminant validity by comparing several relevant subgroups in our sample (based on age, depression, IADL dependency, living situation, etc.). We also investigated the relationship between overall wellbeing and the ICECAP-O, controlling for HrQol and background characteristics.

**Results:**

This study suggests that the ICECAP-O has good convergent validity with wellbeing measures as well as health measures and discriminates between various groups of post-hospitalized older people. Wellbeing measured by both Cantril’s ladder and SPF-IL is associated with the ICECAP-O in a multivariate analysis controlling for HRQoL as well.

**Conclusion:**

The ICECAP-O seems to be a valid instrument of capability-wellbeing in older, post-hospitalized people, showing good convergent validity with health and wellbeing instruments, and is able to discriminate between elderly with various health profiles. The ICECAP-O measure seems to capture both health and wellbeing. Therefore it is a promising instrument for assessing the outcomes of health and social services aimed at older people.

## Background

Economic evaluation of healthcare services aims to inform policy makers by comparing the costs and benefits of alternative health care interventions. In such an evaluation, it is crucial that besides all costs, all benefits of healthcare services are captured. Capturing such benefits can be challenging, since healthcare services such as elderly care, long-term mental health, and public health may impact individuals health and health related quality of life, as well as their wellbeing more generally [1-4].

Health can be defined as a multidimensional construct of physical, psychological and social dimensions [5]. These health dimensions can be inter-related, for example decreased mobility may lead to a decrease in social contacts and depression [6,7], subsequently impacting social and psychological dimensions of health [7]. Health related quality of life (HrQol) tries to capture how health impacts individuals’ Quality of Life (Qol) [8]. In economic evaluations, benefits are frequently assessed by changes in health-related quality of life combined with the duration an individual spends in various health states. Duration and HrQol are then subsequently combined in Quality-Adjusted Life-Years (QALYs), and thus arguably capture the effect of healthcare services on physical, psychological and social dimensions of health. Aspects of broader wellbeing, such as maintaining independence, dignity, and comfort [1], however, arguably are not captured by the concept of HrQol in its entirety. This can cause problems in capturing the full benefits of interventions, in particular in the evaluation of social care interventions, as well as integrated health and social care services [9]. For example, specific social care interventions like day care and meals on wheels may improve wellbeing, but not health, or at least not only health [9]. As a consequence, such services cannot be evaluated in the same manner as other healthcare services such as medicines [9] where using HrQol seems more appropriate in many cases. Otherwise, the benefits of these provisions may be undervalued [10].

Therefore, broadening the evaluative space of economic evaluations by a wider measurement of benefits has been suggested in evaluation of elderly care [1,11], using dimensions of wellbeing such as independence, attachment, or the ability to pursue valued activities [10] in addition to health dimensions. In that context, a proposed alternative to measuring HrQol is to measure capabilities. Capabilities may be seen as a conceptualization of wellbeing [1], defined as the capacity to perform certain actions and achieve certain states (irrespective of actually doing so). Capability wellbeing assesses what individuals can do instead of focusing on functioning, i.e. what individuals actually do [1]. Capability-wellbeing captures a variety of health and non-health dimensions, which may be difficult to separate [12].

In order to measure capability wellbeing, two instruments have been developed to date, the ICECAP-O [10,13] (ICEpop (Investigating Choice Experiments for the Preferences of Older People)) CAPability measure for Older people above 65 and the ICECAP-A [1] for the general population. Both instruments are intended as outcome measures for economic evaluations of both health and social services, where beyond health, wellbeing aspects have to be considered as well [1,9,10]. In order to be useful for economic evaluations, instruments should be sufficiently validated in terms of their convergent and discriminant validity. While the ICECAP-A has been validated in the UK only [14], the ICECAP-O has been validated in a number of settings: in the British general elderly population [10], in an Australian population of post-hospitalized elderly receiving residential care [15], in a Canadian population of elderly visiting a fall-prevention clinic [16] and a proxy version has been validated in Dutch nursing home settings [17].

However, to date, the ICECAP-O has not been validated in a population of post-hospitalized older-people in the Netherlands. Post-hospitalized elderly are increasingly recognized as a population in which health improvements can be achieved [18] through geriatric interventions. In the Netherlands, in the context of the National Care for the Elderly Program significant efforts are made to improve health and quality of life outcomes in frail elderly, for instance through the Prevention and Reactivation Care Programme among older patients who are admitted to a hospital [19]. For elderly populations, hospitalization increases the risk of functional decline, defined customarily as a decrease in (instrumental) activities of daily living ((I)ADL) [20]. Although elderly may be hospitalized due to function decline resulting from illness, such functional decline is also frequent after admission: 35% of 70 year olds and 65% of 90 year olds experience such a decline. Functional decline is therefore influenced by hospital care as well [20], through increased complications [21] or through less aggressive treatment regimens than customary in younger populations [18]. In a group of post-hospitalized older people, a wide range of differences in health, capabilities and well-being problems may be expected due to (differences in) age, physical function, and other characteristics of the elderly such as multi-morbidity and support from their direct environment. As a result, this population is likely to receive various forms of publicly funded healthcare, as well as being the recipients of other social services. Furthermore, there is little research on how the ICECAP-O is related to other conceptualizations of wellbeing and the relationships between the ICECAP-O and measures of health (physical, psychological and social) remain underexplored. Exploring such issues is preferably done in a group in which a variety of health and well-being problems may be expected such as post-hospitalized elderly. Therefore, the aim of this study is to validate the convergent and discriminant validity of the ICECAP-O in a Dutch community-dwelling population discharged from a hospital in the prior three months. We further study the discriminant validity of the ICECAP-O by performing sub-group analyses, highlighting the differences in ICECAP-O scores between groups of elderly.

## Method

### Design, participants and setting

This validation study was based on a pilot study of the Transition-experiment Geriatric Network Rotterdam Prevention and Reactivation of Care program. The aim of the pilot was to select outcome measures and triage instruments for the actual trial [19]. In order to be able to select appropriate instruments, several instruments measuring similar constructs were included in the pilot. As some instruments such as the ICECAP-O were not widely validated, their validity was further examined on the basis of the pilot. This helped to reduce the number of instruments measuring the same concepts in the actual trial. This pilot study was conducted among all older people admitted to the Vlietland hospital between June and October 2010. The sample included 500 older people (>65 years of age) who were interviewed using face to face questionnaires. Three months after hospital admission, a total of 296 discharged patients (59% response rate) completed questionnaires using face to face administration and were included in the analysis. Reasons for dropout were: death (n=49), lost interest to participate (n=52), too ill (n=35), terminally ill (n=5), objection by partner/family (n=14), mentally not able (n=8), private reasons (e.g. death of spouse; n=4), questions not applicable (n=8), no contact/unable to reach respondent (n=12), and reason unknown (n=22). The study protocol was approved by the medical ethics committee of the Erasmus Medical Centre, Rotterdam, the Netherlands, under protocol number MEC2011-041. Informed consent was obtained from all participants. The study protocol is extensively described in Asmus-Szepesi [19].

### Measures

To investigate the convergent and discriminant validity of the ICECAP-O, we used a wide variety of outcome measures. To measure different conceptualizations and operationalizations of wellbeing we used three wellbeing measures. First, capability wellbeing was measured using the ICECAP-O capability measure for older people. The ICECAP instruments can be seen as measuring capability wellbeing [1] achieved by the capacity to perform certain actions and achieve certain states [9]. The ICECAP-O measures five capability dimensions – attachment, security, role, enjoyment, and control – with one question per dimension. Each dimension can be scored on four levels, thus distinguishing 1024 possible ‘capability states’. The ICECAP-O was developed using rigorous qualitative and quantitative approaches [9,10,13,22]. In order to obtain tariffs for the ICECAP-O, the attributes were valued using best-worst scaling, a special type of discrete choice analysis [9]. The ICECAP-O tariffs have values between 0 (no capability) and 1 (full capability). Second, wellbeing was measured using the Cantril’s ladder life satisfaction scale, a one-dimensional index ranging from zero (completely dissatisfied) to 10 (completely satisfied) [23]. Third, we also used a multi-dimensional measure of wellbeing, the Social Production Function: Instrument for Level of Well-being (SPF-IL), to assess wellbeing. The SPF-IL measures affection, behavioral confirmation, status, comfort and stimulation on a 4 point scale, ranging from 1 (never) to 4 (always) [24], providing an overall index of wellbeing, with higher scores indicating higher levels of wellbeing.

To measure HRQol we used the EQ-5D [25]. The EQ5D measures HrQol in terms of five dimensions (mobility, self-care, daily activities, pain and discomfort, anxiety and depression) with three levels each (1=no problems, 2=moderate problems, and 3=extreme problems) describing 243 health states. The EQ-5D health states can be converted into a utility score by applying the scoring values (tariff) for the Dutch population [25]. The EQ5D utility scores range from 1 (perfect health) through 0 (death) and has negative values accounting for health states worse than dead [25]. The EQ5D is one of the most widely used measures of HrQol, and is extensively used in economic evaluations [25]. To assess physical functioning, we used the combined ADL (Activities of Daily Living)-IADL (Instrumental Activities of Daily Living) scale (Katz-15) consisting of yes or no responses on IADL items such as bathing, dressing and abilities such as using the telephone and managing money [26]. The IADL scores range from 0–15 with higher scores indicating higher dependency. Three cutoff-scores are commonly used, 7 (severely IADL dependent), 4 (moderately IADL dependent) and 1 (mildly dependent) [27]. In this current study we used the cutoff score for mildly dependent.

To assess depressive symptoms, we used the Geriatric Depression Scale-15 (GDS-15). The GDS-15 consists of 15 items, measuring psychological function and mood swings. The instrument has been widely validated in older people [28]. The cutoff score of 10 is a reliable cut-off score for major depression, while a score below five is considered to indicate the absence of clinically significant depressive symptoms. Scores between 5 and 10 indicate mild depression [29,30]. In this current study we used the cutoff score of five.

To assess social functioning, we used the social activity limitation item from the SF-20 [31]. This item measures the frequency with which respondents experienced social activity limitations due to health. The item runs from 1 (none of the time) to 6 (all of the time), and converts to a 0–100 scale. In this current study we have used a cutoff score at the middle of the scale, i.e. 50, to distinguish elderly who have frequent limitations (limitations a good bit of the time or more frequently) from those with less frequent limitations.

Finally, we investigated the presence of multi-morbidity. Multi-morbidity was defined as having two or more chronic disease conditions, as is common in the literature [32,33]. We included the following chronic illnesses in our multi-morbidity count: diabetes, stroke (cerebral haemorrhage, cerebral infarction or TIA), heart failure, cancer (malignant condition), asthma or chronic bronchitis or lung emphysema or COPD, incontinence, degenerative arthrosis of hip or knee, osteoporosis, prostate symptoms caused by benign prostate enlargement, dementia, hearing problems, problems with vision.

### Hypotheses

For convergent validity, we expect the ICECAP-O capability wellbeing measure to correlate more strongly with Cantril’s ladder and the SPF-IL wellbeing measures, than with the EQ5D HrQol measure and with the IADL, GDS and the SF-20’s social activity limitation health measures, because the ICECAP-O is intended as measure of well-being that transcends measuring HrQol [13]. For discriminant validity, we expect to find higher ICECAP-O scores in older people living with others as compared to living alone due to higher affection [10,34]. We also expect to find higher scores in IADL independent as compared to IADL dependent older people, and for non-depressed as compared to depressed older people as well as in older people with no social activity limitations vs. those with such limitations. This was based on earlier work showing strong relationships between the ICECAP-O role, enjoyment and control dimensions and physical problems, and between the ICECAP-O dimensions attachment and enjoyment and mental health measures, and between a number of social measures and the ICECAP-O dimensions role and enjoyment [10]. Furthermore, we will explore differences on the ICECAP-O in older people living at home compared to those in a nursing home, in the young-old (<75 years old) compared to the old-old (≥75 years old) and in multi-morbid older people versus those without multi-morbidity (the latter expected to score higher on the ICECAP-O). In order to gain further insight into how the ICECAP-O and health are related to older and more accepted wellbeing measures, we will explore if the ICECAP-O is related to other measures of wellbeing in a multivariate model controlling for health.

### Analysis

All analyses were performed in STATA 11. Item level analysis of non-response was carried out. For all analyses, available cases were used.

We calculated descriptive statistics. In establishing convergent validity we used correlation analyses. Correlations above 0.5 are referred to as strong, between 0.3 and 0.5 as moderate, and below 0.3 as weak. Differences in strength of correlation between ICECAP-O and EQ5D, and between ICECAP-O and the wellbeing measures were assessed with Steiger’s Z [35]. For discriminant validity we used t-tests for two group comparisons and one-way ANOVA for comparisons between multiple groups. To further explore discriminant validity of the ICECAP-O, we also performed stepwise regression analyses with a p-value of 0.2. To analyze to what degree the ICECAP-O is related to the Cantril’s ladder and SPF-IL wellbeing measures, we have performed stepwise multivariate regressions including all variables with a p-value below 0.2. Regression assumptions were checked. In the subgroup analysis, categorical groups were compared using chi-squared tests.

## Results

### Response

296 clients completed face to face questionnaires three months after admission, and were included in the analysis. For these included clients, demographic characteristics had no missing values, while for other variables missing values ranged from 2 (0,7%) in case of Cantril’s ladder to 12 (4%) in case of the ICECAP-O tariffs. Response on the ICECAP-O dimensions was quite good, ranging from 97% on the role dimension to 99% on the control dimension, demonstrating good feasibility. All analyses below were conducted on a net sample using complete case analysis (n=275).

**Figure 1:**
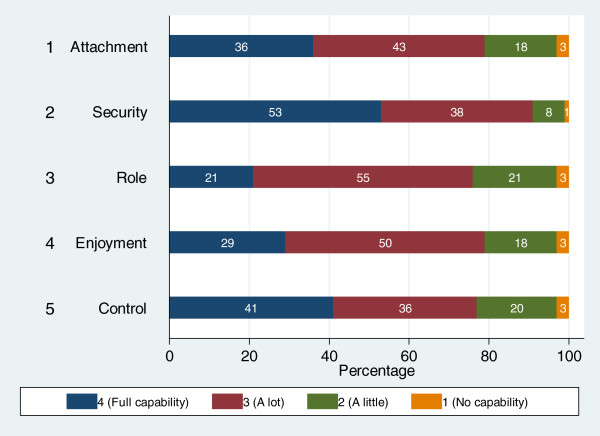
Response on the ICECAP-O.

Table 1 shows the demographic characteristics of post-hospitalized elderly, as well as their health status, HRQol, and wellbeing. Figure 1 below details the response to the individual ICECAP-O dimensions.

**Table 1:** Sample characteristics

***Variable***	***Complete-case analysis (n=275)***	***Mean (SD)***	***Percentage***
Age	Young old (65–75)	76.21 (6.79)	46.55
	Old-old (75+)		53.45
Sex	Female		53.82
	Male		46.28
Education	None		6.55
	Primary school		26.90
	Lower vocational		18.18
	General secondary education		34.18
	Grammar school		9.09
	Polytechnic/higher vocational education/University		5.09
Maritial Status	Married/Other living together		57.46
	Divorced		5.82
	Widow(er)		30.90
	Never married		5.82
Living arrangement	Home alone		37.09
	Home with partner or children		56.73
	Nursing home/elderly home		6.18
Diagnoses at admission	Diabetes		20,96%
	Stroke, cerebral haemorrhage (bleed in the brain), cerebral infarction (blocked blood vessel in the brain) or TIA		9,97%
	Heart failure		38,49%
	A type of cancer (malignant condition)		16,49%
	Asthma, chronic bronchitis, lung emphysema or COPD		22,68%
	Incontinence		20,27%
	Degenerative arthritis of hip or knee		49,48%
	Osteoporosis		27,49%
	Hip fracture		5,50%
	Other fractures		9,97%
	Dizziness with falling		16,15%
	Prostate symptoms caused by benign prostate enlargement		8,59%
	Depression		7,56%
	Anxiety/panic disorder		4,12%
	Dementia		0,69%
	Hearing problems		23,37%
	Problems with vision		15,81%
Multimorbidity	Maximum 1 chronic condition		34.55
	Multimorbid (more than 2 chronic conditions)		65.45
ICECAP-O tarrifs		0.84 (0.14)	
Cantril’s ladder		7.43 (1.32)	
SPF-IL		2.85 (0.43)	
EQ5D	Mobility –some problems		49.45
	Self-care –some problems		11.64
	Self-care – severe problems		2.18
	Daily activities		25.45
	Daily activities – severe problems		5.09
	Pain and discomfort – some problems		40.73
	Pain and discomfort- severe problems		8.00
	Anxiety and depression – some problems		12.00
	Anxiety and depression – severe problems		0.73
	EQ5D utilities	0.80 (0.17)	
Health measures	SF-20 social activity limitations	74.18 (26.18)	
	GDS	2.55 (2.61)	
	IADL (average dependency)	2.47 (2.59)	

### Convergent and discriminant validity

Correlation analysis shows, that the ICECAP-O overall tariffs were significantly and strongly correlated with Cantril’s ladder, while the ICECAP-O dimensions were generally moderately correlated with Cantril’s ladder. The SPF-IL total scores were generally moderately correlated with the ICECAP-O dimensions and strongly correlated with the ICECAP-O tariffs. The overall EQ5D utility score was also moderately correlated with the ICECAP-O tariffs. The EQ5D dimensions were mostly weakly correlated with the ICECAP-O tariffs, with the exception of Self-Care and Control, Usual activities and Role, and Usual activities and Control for which moderate correlations were found. Correlations between other health measures and the ICECAP-O tariffs were generally moderate, with the correlation between GDS and Attachment being weak. GDS and IADL were both strongly correlated with the ICECAP-O tariffs. The social activity limitations dimension was moderately correlated with Role, Enjoyment, Control and the ICECAP-O tariffs. Using Steiger’s Z, we found that the difference in strength of the correlation between the ICECAP-O and the wellbeing measures on the one hand and between the ICECAP-O and the EQ5D on the other hand was not statistically significant (Table 2).

**Table 2:** Correlations between capability, wellbeing and health dimensions

	***ICECAP-O capability dimensions***					***Weighted capabilities***
	**Attachment**	**Security**	**Role**	**Enjoyment**	**Control**	**ICECAP-tariffs**
*Wellbeing*						
Cantril’s ladder	0.31**	0.22**	0.46**	0.46**	0.28**	0.51**
SPF_IL	0.47**	0.27**	0.43**	0.48**	0.34**	0.60**
*Health*						
EQ5D Mobility	−0.17**	−0.08	−0.35**	−0.20**	−0.32**	−0.30**
EQ5D Self-care	−0.16**	−0.12	−0.35**	−0.25**	−0.42**	−0.39**
EQ5D Usual Activities	−0.17**	−0.19**	−0.47**	−0.31**	−0.43**	−0.47**
EQ5D Pain/Discomfort	−0.13	−0.13*	−0.28**	−0.25**	−0.25**	−0.25**
EQ5D Anxiety/Depression	−0.07	−0.25**	−0.18**	−0.30**	−0.16**	−0.25**
EQ5D utilities	0.12*	0.20**	0.40**	0.30**	0.40**	0.40**
SF-20 social activity limitations	0.19**	0.22**	0.46**	0.34**	0.42**	0.47**
GDS	−0.29**	−0.35**	−0.42**	−0.46**	−0.36**	−0.57**
IADL	−0.24**	−0.16*	−0.47**	−0.31**	−0.60**	−0.51**

* p value<0.05 ** p-value <0.01.

Results regarding discriminant validity of the ICECAP-O are shown in Table 3. In the bivariate analysis the ICECAP-O significantly discriminated between young-old and old-old, between multi-morbid and single-morbid respondents, depressed and non-depressed respondents, between IADL dependent and non dependent respondents as well as between respondents with frequent social activity limitations and those without. Furthermore, the ICECAP-O discriminated between people with higher and lower EQ5D scores. This is similar to the other wellbeing instruments as shown in Table 4, although only the ICECAP-O discriminated the young-old and the old-old. In the multivariate stepwise regression, the ICECAP-O discriminated groups based on IADL dependency, depressive symptoms, social activity limitations and EQ5D scores (operationalized as dummies).

**Table 3:** Discriminant validity of the ICECAP-O in select groups

***Variable***	***Level***	***ICECAP-O***			
		**Bivariate group comparisons**		**Multivariate group comparisons (stepwise regression)**	
**Demographic**		**Mean**	**p-value**	**Standardized coefficients**	**p-value**
Age	Older people below 75	0.86*	0.01		
	Elderly above 75	0.83			
Sex	Female	0.85	0.35		
	Male	0.84			
Education	Pre-secondary	0.86	0.10		
	Post-secondary	0.83			
Married	Married or other living together	0.85	0.13		
	Divorced	0.76			
	Widow	0.85			
	Never married	0.83			
Living situation	Alone	0.84	0.13		
	With partner/children	0.84			
	Nursing home	0.78			
Health					
Multimorbid	Maximum 1 chronic condition	0.89**	0.00		
	More than 2 conditions	0.82			
IADL	Independent	0.92**	0.00	−0.21**	0.00
	Dependent	0.81			
SF-20 social activity limitations	No limitations	0.90**	0.00	−0.27**	0.00
	Limited	0.77			
GDS	Not depressed	0.88**	0.00	−0.29**	0.00
	Depressed	0.73			
EQ5D	Top 50%	0.90**	0.00	0.13	0.01
	Bottom 50%	0.80			
R squared					0.38

* p-value <0.05 ** p-value<0.01.

**Table 4:** Comparison of the discriminant validity of the wellbeing instruments

**Variable**	**Level**	**Cantril’s ladder**	**SPF-IL**	***ICECAP-O***
**Demographics**		**Mean Cantril’s ladder score**	**Mean SPF-IL score**	**Mean ICECAP-O score**
Age	Older people below 75	7.51	2.86	0.86*
	Elderly above 75	7.36	2.81	0.83
Sex	Female	7.44	2.82	0.85
	Male	7.41	2.84	0.84
Education	Pre-secondary	7.49	2.89	0.86
	Post-secondary	7.36	2.77	0.83
Married	Married or other living together	7.58	2.86	0.85
	Divorced	6.13	2.60	0.76
	Widow	7.38	2.86	0.85
	Never married	7.56	1.84	0.83
Living situation	Alone	7.17	2.80	0.84
	With partner/children	7.67	2.87	0.84
	Nursing home	6.76	2.66	0.78
Health				
Multimorbid	Maximum 1 chronic condition	7.76**	2.99**	0.89**
	More than 2 conditions	7.26	2.75	0.82
IADL	Independent	7.99**	3.01**	0.92**
	Dependent	7.17	2.74	0.81
SF-20 social activity limitations	No limitations	7.78**	2.97**	0.90**
	Limited	6.92	2.63	0.77
GDS	Not depressed	7.73**	2.95**	0.88**
	Depressed	6.48	2.47	0.73
EQ5D	Top 50%	7.87**	2.97**	0.90**
	Bottom 50%	7.10	2.72	0.80

Indicating bivariate significance: * p-value <0.05 ** p-value<0.01.

### Subgroups

Differences in demographic characteristics between the population with the highest ICECAP-O scores (highest third, n=111) and the lowest ICECAP-O scores (lowest third, n=94) were also investigated (analysis not shown here). Significant differences were found for age (older people having lower ICECAP-O scores), place of residence (living in a nursing home being associated with lower scores) and multi-morbidity (which is associated with lower scores). As for the other measures, a low ICECAP-O score is significantly associated with lower Cantril’s ladder scores, SPF-IL scores and EQ5D scores. As for GDS and IADL, depressed respondents and those with functional limitations were more likely to be in the group with low ICECAP-O scores.

### Relationship between the ICECAP-O and measures of overall wellbeing

In a multivariate analysis of other measures of wellbeing, capability wellbeing as measured by the ICECAP-O tariffs was significantly and positively associated with wellbeing as measured by Cantril’s ladder and the SPF-IL. HrQol as measured by the EQ5D utility scores was not independently associated with SPF-IL or Cantril’s ladder after ICECAP-O tariffs were included in the regression analyses. Being depressed was independently associated with lower Cantril’s ladder as well as SPF-IL scores. Marital status and living arrangement were significantly related to Cantril’s ladder but not to SPF-IL. Multimorbidity was associated with lower SPF-IL scores, but not significantly associated with Cantril’s ladder scores (Table 5).

**Table 5:** Stepwise regression between Cantril’s ladder, ICECAP-O and health variables*

	***Dep: Cantril’s ladder***		***Dep: SPF-IL***	
	**Standardized regression coeff**	**p-value**	**Standardized regression coeff**	**p-value**
**ICECAP-O tariffs**	0.26	0.00	0.35	0.00
**EQ5D utilities**			0.07	0.17
**GDS**	−0.38	0.00	−0.36	0.00
**Divorced**	−0.06	0.48		
**Widow**	0.15	0.03		
**Never married**	0.07	0.11		
**Living alone at home**	0.25	0.00		
**Living in a nursing home**	−0.05	0.48		
**Multimorbidity**			−0.10	0.04
**Adj. R-square**		0.41		0.46

*Demographic variables have been converted to dummies, and inserted in batch.

## Discussion

### Summary of main results

As hypothesized, the capability wellbeing instrument ICECAP-O tariffs were significantly correlated with other measures of wellbeing (Cantril’s ladder, the SPF-IL) as well as with all health measures (EQ5D dimensions and utilities, IADL, GDS, SF-20 Social Activity limitation). Contrary to expectations based on the type of instrument, the strength of the correlation between the ICECAP-O and the wellbeing measures was fairly similar as that with health measures. The individual ICECAP-O dimensions were also correlated with the overall scores of the different health and wellbeing measures. Overall, we found significant correlations between the ICECAP-O dimensions and the individual EQ5D dimensions, with the exception of Attachment, which was not significantly correlated with the Pain/Discomfort and Anxiety/Depression dimensions of the EQ5D and Security, which was not significantly correlated with the EQ5D dimensions Mobility and Self-care. As hypothesized, the ICECAP-O discriminated between the following measures in the bivariate and multivariate analyses: depressed and non-depressed elderly, IADL dependent and non IADL dependent elderly and between those with social activity limitations and without social activity limitations. In the exploratory analysis the ICECAP-O discriminated between multi-morbid and other elderly and between elderly with high and low EQ5D scores. Regarding measures of wellbeing, the ICECAP-O is significantly related to both Cantril’s ladder and the SPF-IL, even when correcting for health variables.

### Limitations

This study has a number of limitations worth mentioning. First, our sample of elderly was not representative, but consisted of post-hospitalized elderly, who were previously admitted to a single hospital, living in one region of the Netherlands. Elderly in our sample are frailer than the general community-dwelling elderly population, reporting lower levels of mobility on the EQ5D [36-38] than customary for the age group. Such reduced mobility suggests that our population is characterized by functional decline, consistent with frailty. In addition, patients in our sample were characterized by a broad range of diseases and multiple chronic conditions, with heart failure and osteoporosis being the most common diagnoses. Such a relative high number of elderly with multi-morbidity is also consistent with frailty. Associations between capabilities, health and well-being may be weaker in a general sample of frail elderly due to less variation in measurements. However, we have no indication that the selection of respondents drives the results regarding validity. Future research in other community-dwelling elderly populations also in other countries than the UK is necessary to further test this and validate the instrument. Second, we used a stepwise regression to identify explanatory variables of the ICECAP-O scores, which has limitations. In order to avoid rejecting possible significant variables, we used a relatively high p-value (0.2) for excluding variables. Additionally, we performed a regression analysis with all possible independent variables, which confirmed the results from the stepwise regression. It is worth noting moreover that, given the modest sample size, some subgroups were relatively small. This may lead to lack of power in establishing significant relationships.

### Comparability with other findings

Compared to previous studies [10,15-17,34], the values for the individual dimensions and overall scores of the ICECAP-O in this current study are similar to those obtained in the general elderly population and substantially higher than those obtained in a Dutch nursing home [17]. The current scores are comparable to the British and Australian reference values [10,15,34], with the exception of the attachment dimension, where the British and Australian studies [10,15,34] report a higher percentage of older people at full capability (57% British and Australian studies vs. 36% current study) and the security dimension, where this current study has a far higher percentage of older people at full capability (53% current study vs. 18% British study vs. 37% Australian study). The differences in the attachment dimension cannot be explained by differences in the fraction of married elderly, which is quite similar across the studies. However, the elderly in the current study are a worse-off group (i.e. in terms of mobility) than the general elderly population in the UK, which may partly explain the lower scores on the attachment dimension. Differences on the security dimension may be explained by cultural differences in answering this question. Indeed, this is the second study in the Netherlands in which relatively high scores were found for the security dimension [17]. Hence, Dutch elderly either have fewer concerns about the future than UK elderly or are less likely to share their concerns about the future. It also seems important to further investigate whether the translation of the description of the security dimension may lead to the observed differences. The average overall scores found here i.e. 0.84 were comparable to those obtained in the British and Canadian population (0.82), the Australian population (0.81) and substantially higher than for older people in a nursing home (0.63). Comparison of the overall scores suggest that on average the ICECAP-O scores of the Dutch community-living elderly are comparable to the general population in Australia and the UK, and are substantially better than elderly living in nursing homes in the Netherlands.

Furthermore, the correlations between the ICECAP-O, Cantril’s Ladder and EQ5D show broadly similar results as reported in previous studies, with a number of exceptions. Unlike the British validation study [10] but in line with the Australian study [15], we found a statistically significant though moderate correlation between ICECAP-O attachment dimension and the EQ5D dimensions mobility, self-care and usual activities. In addition, unlike the British study we found a significant correlation between the ICECAP-O’s security dimension and the EQ5D dimensions usual activities and pain. It must be noted that these are quite weak correlations, and significance may or may not be reached due to minor differences in sampling variation. Such minor differences in sample variation may be related to differences in the respective samples; here we approached previously hospitalized elderly, while the British study was performed in a sample from the general elderly population. Our correlation results were also comparable to a Dutch study using proxy respondents in nursing homes [17]. There, however, the correlation between the ICECAP-tariffs and the EQ5D was somewhat stronger then found here, which may be due to differences between self-report and proxy responses. In this study the ICECAP-O is unrelated to Sex and Education level, which is consistent with previous findings.

### Relationship between health and wellbeing and the ICECAP-O

Comparing the performance of the ICECAP-O to that of other health and wellbeing instruments, some aspects deserve mentioning. Given the strong correlations between the ICECAP-O measure of capability wellbeing and the other two wellbeing measures, as well as between the ICECAP-O measure and the EQ5D HrQol measure, ICECAP-O scores are related to both health and other wellbeing scores. The ICECAP-O scores are moreover related to individual health dimensions in terms of physical functioning, psychological functioning and social functioning. The tests of discriminant validity confirm this relationship between health measures and the ICECAP-O scores. Even though the ICECAP-O does not have an explicit physical dimension [39], it seems that it is capable of capturing the effect of decreased physical function on capability wellbeing to a large degree, primarily through the control and role dimensions. With respect to the wellbeing instruments, the strong correlation between the ICECAP-O and Cantril’s ladder as well as the SPF-IL suggests that the ICECAP-O is related to these wellbeing measures as well, which is also confirmed in multivariate analyses. Table 4 does suggest that GDS has an influence on SPF-IL and Cantril’s ladder beyond what is captured by the ICECAP-O. This may be related to the concept of capability wellbeing or to the ICECAP-O instrument’s insensitivity for depression.

### Implications for policy and future research

The ICECAP-O is a measure of wellbeing, and therefore has the potential to broaden the evaluative space of economic evaluations in health care by focusing on more than health alone. As such, it can potentially compare the benefits across a large number of sectors which (primarily) aim to improve wellbeing, such as (parts of) social care [2], institutionalized elderly care [40], public health [3], and mental health [4]. This is a particularly useful property in case of populations such as frail elderly characterized by decreasing independence and multi-morbidity, potentially across different health dimensions. The ICECAP-O measures (one conceptualization of) wellbeing. In doing so, its outcomes are, expectedly, related to health outcomes. The ICECAP-O moreover discriminates between various better off and worse-off groups. In this current study, in a post-hospitalized group significant insights were gained in terms of the relationship between capability wellbeing, life satisfaction, SPF_IL and various health measures. On the basis of our findings, we advocate the further use of the ICECAP-O measure in the context of economic evaluations, especially in those circumstances where broader well-being effects are expected and in combination with other measures. It can also be used in large scale surveys aimed at identifying depraved populations in order to identify groups which may benefit from interventions, as has been done previously [34]. Nonetheless, a number of issues need to be explored further.

Further research is required to confirm the current favorable findings and to further explore the feasibility, validity and usefulness of the ICECAP-O instrument, also in the context of economic evaluations. In that context, larger studies would be helpful, allowing more subgroup analyses, as well as studies in different contexts (e.g. specific disease areas, living environments or cultural settings). Further research is especially encouraged in more homogeneous population characterized by a single disease. Furthermore, since the performance of the ICECAP-O has not been widely explored in longitudinal studies, the sensitivity to changes of the ICECAP-O is currently unclear. Whether the ICECAP-O comprehensively captures health and wellbeing changes, including depression, also deserves further attention. Additionally, further research is necessary to establish a causal relationship between health and wellbeing as measured by the ICECAP-O, and to explore ways in which the capabilities of older people can be improved.

## Conclusion

The ICECAP-O is an outcome measure which may be particularly useful in the context of (economic) evaluations of health care services such as long-term elderly care, where broader effects are expected than those captured with conventional HRQoL measures. In the current study, the ICECAP-O showed good convergent validity with validated measures of health and well-being as well as good discriminant validity in a heterogeneous population of post-hospitalized elderly. As such, the ICECAP-O seems to be a promising instrument. Additional research is required to not only confirm these findings in other settings and samples, but also to study the sensitivity to change of the instrument as well as its comprehensiveness in all relevant wellbeing effects.

## Abbreviations

HrQol: Health-related quality of life; Qol: Quality of Life; QALY: Quality-adjusted life years; ICECAP-O: ICEpop (Investigating choice experiments for the preferences of older people); SPF-IL: Social production function- Instrument for level of well-being; IADL: Instrumental activities of daily living; GDS: Geriatric depression scale.

## Competing interests

The authors declare that they have no competing interests.

## Authors’ contribution

All authors made substantial contribution to the design, analysis, interpretation of data, have been involved in drafting and revising the manuscript and have approved the final version.
